# The Effect of Virtual Reality on Preoperative Anxiety: A Meta-Analysis of Randomized Controlled Trials

**DOI:** 10.3390/jcm9103151

**Published:** 2020-09-29

**Authors:** Chang-Hoon Koo, Jin-Woo Park, Jung-Hee Ryu, Sung-Hee Han

**Affiliations:** 1 Department of Anesthesiology & Pain Medicine, Seoul National University Bundang Hospital, Seongnam 13620, Korea; vollock9@gmail.com (C.-H.K.); jinul8282@gmail.com (J.-W.P.); 2 Department of Anesthesiology & Pain Medicine, Seoul National University College of Medicine, Seoul 03080, Korea

**Keywords:** preoperative anxiety, surgery, virtual reality

## Abstract

Virtual reality (VR), a technology that provides a stimulated sensory experience, has recently been implemented in various fields of medicine. Several studies have investigated the efficacy of VR on preoperative anxiety. The purpose of this meta-analysis was to validate whether VR could relieve preoperative anxiety in patients undergoing surgery. Electronic databases were searched to identify all randomized controlled trials (RCTs) investigating the effect of VR on preoperative anxiety. The primary outcome was defined as the preoperative anxiety scores. We estimated the effect size using the standard mean difference (SMD) with a 95% confidence interval (CI) using a random effect model. Ultimately, 10 RCTs, with a total of 813 patients, were included in the final analysis. Preoperative anxiety was significantly lower in the VR group than in the control group (SMD −0.64, 95% CI −1.08 to −0.20, *p* = 0.004). In a subgroup analysis, the preoperative anxiety scores were lower in the VR group than in the control group in pediatric patients (SMD −0.71, 95% CI −1.14 to −0.27, *p* = 0.002), whereas a significant difference was not observed between the two groups in adult patients (*p* = 0.226). The results of this meta-analysis indicated that VR could decrease preoperative anxiety, especially in pediatric patients.

## 1. Introduction

Surgery is a challenging and stressful event, and about 80% of patients scheduled for surgery experience preoperative anxiety due to fear of pain, fear of complications, or fear of death [1,2,3]. Preoperative anxiety causes physiologic and psychological changes by releasing catecholamine and inducing cardiovascular responses, such as tachycardia, hypertension, and arrhythmia [3,4]. It can also increase anesthetic needs, postoperative pain, and analgesic requirements [5,6]. Hence, relieving preoperative anxiety can improve the quality of recovery in patients undergoing surgery.

Virtual reality (VR) is a computer technology that provides an immersive experience in a 3 dimensional simulated world by allowing the users to interact with a virtual environment [7]. As made evident by several previous studies, VR has emerged as a viable intervention method in various fields of medicine, especially in relieving pain and anxiety. The effect of VR on anxiety could be explained by two mechanisms—exposure and distraction. Patients can be exposed to the virtual operating room and experience the surgical environment in advance through VR. This allows patients to become familiar with fearful situations [8,9]. Patients may recognize that their imaginary fears are unreal [10]. A previous meta-analysis showed that VR was effectively used as exposure therapy in treating anxiety-related disorders [11]. Distraction is another strategy to relieve anxiety. VR provides attractive materials that can divert a patient’s attention from a stressful situation [12]. Another meta-analysis showed the efficacy of VR as a distraction tool to reduce anxiety during medical procedures [13]. Recently, VR has been applied in the preoperative period to reassure patients, but the benefits of VR on preoperative anxiety remains controversial. Therefore, we designed and conducted a meta-analysis to verify the effect of VR in patients undergoing surgery.

## 2. Materials and Methods

### 2.1. Literature Search

This study was performed in accordance with the Preferred Items for Systematic Reviews and Meta Analyses (PRISMA) statement guidelines [14]. The registration of the protocol is ongoing with the International Prospective Register of Systematic Reviews (PROSPERO). We searched electronic databases (PubMed (National Library of Medicine, Bethesda, MD, USA), EMBASE (Elsevier, Amsterdam, Netherlands), CENTRAL (Cochrane collaboration, London, UK), CINAHL (EBSCO Information Services, Ipswich, MA, USA), Scopus (Elsevier, Amsterdam, Netherlands), Web of Science (Clarivate Analytics, Philadelphia, PA, USA), and KoreaMed (Korean Association of Medical Journal Editors, Seoul, Korea)) to identify studies that evaluated the effects of virtual reality on preoperative anxiety. The search was conducted using MeSH terms and keywords, such as “elective surgical procedure,” “surgery,” “anesthesia,” and “virtual reality” without any restrictions on publication year and publication language. Detailed search strategies for each database are described in Table S1. The literature search was performed up to 28 May 2020.

### 2.2. Study Selection

Two authors (C.-H.K. and J.-W.P.) independently screened and selected the studies according to the predefined inclusion and exclusion criteria. The inclusion criteria were (1) randomized controlled trials (RCTs), (2) included patients who were scheduled to undergo surgery, and (3) patients in the intervention group experienced virtual reality before surgery. The exclusion criteria were (1) non-randomized controlled studies and (2) no full-length articles (e.g., abstract, protocol). Initially, the titles and abstracts of the articles were screened to exclude irrelevant articles. Subsequently, the full texts of articles were reviewed and relevant articles were included in this meta-analysis. If included articles were different between aforementioned authors, another set of authors (J.-H.R. and S.-H.H.) participated in the selection process and made the final decision on study inclusion.

### 2.3. Data Extraction and Risk of Bias Assessment

Two authors (C.-H.K. and S.-H.H.) independently extracted the data from included studies and summarized them into a spreadsheet. The following items were included; author, publication year, sample size, age, preoperative anxiety score, tools for assessing preoperative anxiety, type of surgery, satisfaction, and behavior disturbances. The primary outcome was the preoperative anxiety scores. Secondary outcomes included postoperative satisfaction and behavior disturbances.

Two authors (C.-H.K. and J.-H.R.) independently judged the risk of bias using the Cochrane risk-of-bias tool for randomized trials (RoB) [15]. RoB identifies seven potential sources of bias including random sequence generation, allocation concealment, blinding of participants, blinding of the outcome assessors, incomplete outcome data, selective reporting, and other potential sources of bias. In the present study, the third bias, “blinding of participants,” was omitted because it was impossible to blind patients who received VR before surgery. Consequently, the two authors evaluated six potential risks of bias. Risk of bias was determined to be one of the following three levels: “Low risk of bias,” “Unclear risk of bias,” or “High risk of bias.”

### 2.4. Data Synthesis and Meta-Analysis

Given that the included studies employed various scales to evaluate preoperative anxiety, we estimated the pooled effect size as the standardized mean difference (SMD) and 95% confidence intervals (CI). A random effect model with a standard DerSimonian-Laird approach was used for the analysis due to the anticipated between-study heterogeneity. Mean values and standard deviation (SD) of each group were required to calculate SMD. If the results were presented as the median with range (1st quartile to 3rd quartile or minimum to maximum), we used the Wan’s formula to derive the mean and SD [16]. If the results were presented as other statistics, such as the mean difference with 95% CI, the “effect size calculator” was used to estimate SMD [17]. We performed subgroup analysis based on age of patients (Adult vs. Pediatric patients) because we assumed that VR would be more effective on the latter. We used the I^2^ statistic to identify heterogeneity among studies. According to the Cochrane guidelines [18], it was interpreted as follows: (1) Might not be important (0% to 40%), (2) Moderate (30% to 60%), (3) Substantial (50% to 90%), or (4) Considerable (75% to 100%). We conducted a sensitivity analysis, excluding one study at a time, to determine the robustness of the pooled SMD. Cumulative meta-analysis was performed by sorting the studies based on publication year to explore the chronological trend in the pooled result. Meta-regression was used to identify any potential effect modifiers, such as age, total proportion of female patients, sample size, publication year, or subgroups. A funnel plot was generated to assess potential publication bias, which was to assess a tendency of authors to publish studies with significant results. All statistical analyses were performed using R version 3.6.1 (R Foundation for Statistical Computing, Vienna, Austria) [19] with the “meta” package [20].

## 3. Results

### 3.1. Description of Studies

**Figure 1:**
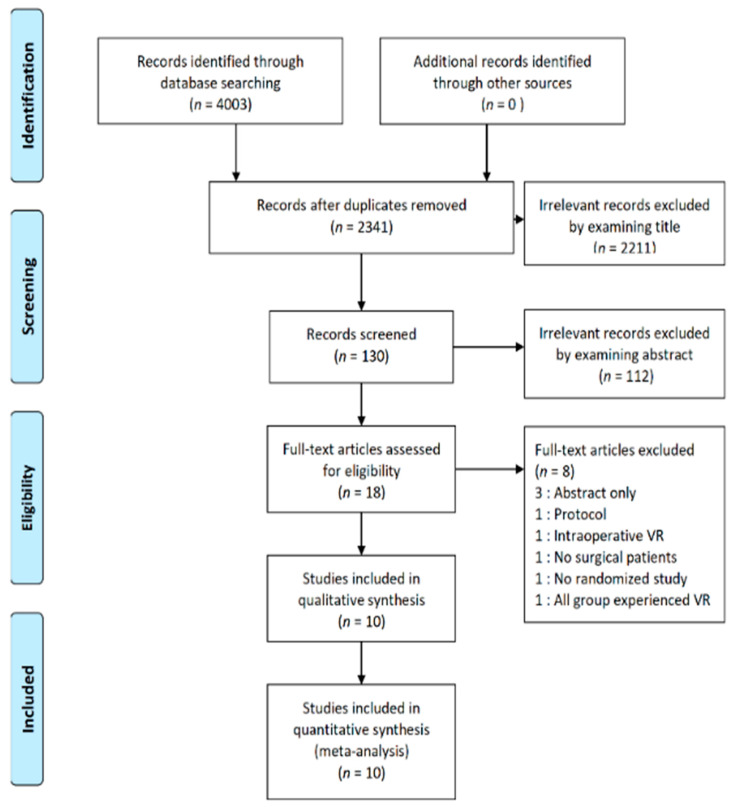
Flow diagram of included and excluded studies.

All of the aforementioned electronic databases provided a total of 4003 articles. Among them, 1662 articles were duplicates and 2323 articles appeared to be beyond the scope of this study based on their titles and abstracts. In the remaining 18 articles, the full texts were examined and among them, eight articles were excluded from the analysis due to the following reasons: no full-length articles (*n* = 4), patients experienced VR during surgery (*n* = 1), healthy subjects (*n* = 1), non-randomized trial (*n* = 1), and patients in both groups experienced VR (*n* = 1). Finally, a total of 10 RCTs were included in this meta-analysis (Figure 1) [21,22,23,24,25,26,27,28,29,30]. Among them, seven studies included two groups (VR group and control group) [21,22,23,27,28,29,30], while three studies included multiple groups [24,25,26]. Of these three studies with multiple groups, two included four groups (two intervention groups and two control groups) [24,25]. According to the Cochrane guidelines [18], we combined two intervention groups into a single VR group and two control groups into a single control group. One study compared the effects among 3-D VR experience, 2-D iPad video, and standard care [26]. We used the data from the VR group and the standard group, excluding the iPad group, which was considered irrelevant within the scope of this study. The detailed information of each study is presented in Table 1.

### 3.2. Preoperative Anxiety Score

The preoperative anxiety score was measured and reported in 10 RCTs, including 813 patients [21,22,23,24,25,26,27,28,29,30]. It was measured with various tools, including modified Yale Preoperative Anxiety Scale [23,27,28,29], Yale Preoperative Anxiety Scale [22], Amsterdam Preoperative Anxiety and Information score [21,30], Hospital Anxiety and Depression Scale [26], Visual Analogue Scale [25], or Numeric Rating Scale [24]. A high score corresponded to a high level of anxiety in 9 RCTs [22,23,24,25,26,27,28,29,30], whereas it was associated with a low level of anxiety in 1 RCT [21]. Therefore, we multiplied the mean values from a given study by “−1” to ensure that all the scales were moving in the same direction, according to the Cochrane guidelines [18]. The preoperative anxiety scores were presented as the means with SD in two RCTs [24,25], median with interquartile range in four RCTs [23,27,28,30], and mean difference with 95% CI between two groups in two RCTs [21,29]. Robertson et al. [26] presented the change in scores, from baseline to post-intervention, without baseline values. Therefore, we estimated the SMD in the anxiety score between the VR group and the control group, imputing a correlation of 0.5 [31]. Dehghan et al. [22] evaluated preoperative anxiety using the Yale Preoperative Anxiety Scale, which consists of four domains. They compared the scores from each domain between groups and only presented the *p*-value of each comparison. Since any numerical data, except *p*-value, was not presented in this study, the SMDs of each comparison were calculated from the *p*-value with sample size and synthesized together for the meta-analysis.

**Figure 2:**
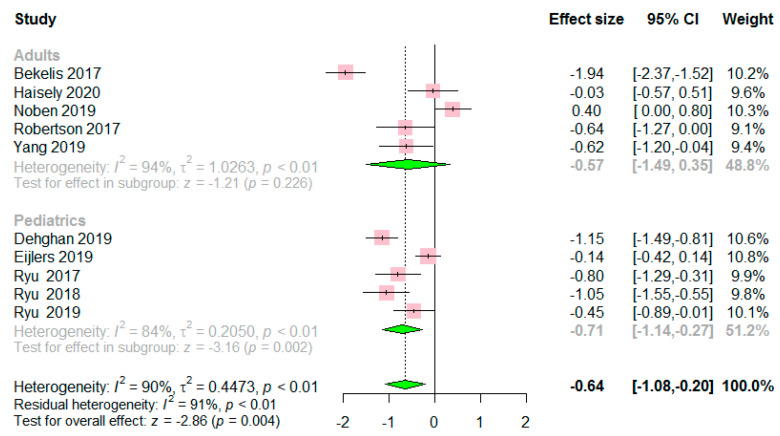
Forest plot comparing the preoperative anxiety score between the VR group and the control group. The preoperative anxiety score was significantly lower in the VR group than in the control group (*p* = 0.004). In subgroup analysis, the preoperative anxiety score was lower in the VR group than in the control group in pediatric patients (*p =* 0.002), while significant difference was not found between the VR group and control group in adult patients (*p* = 0.226).

**Figure 3:**
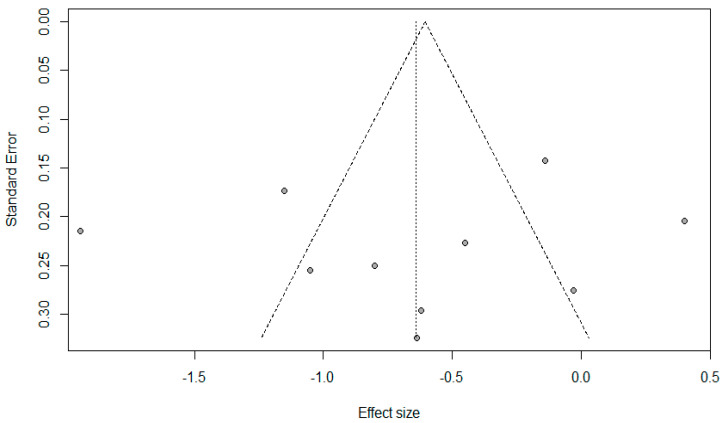
Funnel plot of 10 randomized controlled trials (RCTs) on the effect of VR on preoperative anxiety.

The preoperative anxiety score was significantly lower in the VR group than in the control group (SMD −0.64, 95% CI −1.08 to −0.20, *p* = 0.004) (Figure 2). A considerable level of heterogeneity was found among the RCTs (I^2^ = 90%, *p* < 0.01). Based on the subgroup analysis, the preoperative anxiety score was lower in the VR group than in the control group in pediatric patients (SMD −0.71, 95% CI −1.14 to −0.27, *p* = 0.002), while significant differences in preoperative anxiety were not observed between the VR and control groups in adult patients (SMD −0.57, 95% CI −1.49 to 0.35, *p* = 0.226). Considerable heterogeneity was observed in both pediatric and adult patients (I^2^ = 84%, 94%, respectively). Sensitivity analyses did not change the statistical significance of SMD estimates, confirming the stability of the result (Figure S1). Cumulative meta-analysis showed a trend toward a null effect as data cumulated by year (Figure S2). This implied that the effect of VR has gradually decreased steadily from a large effect in early RCTs. The results of the meta-regression analysis are summarized in Table 2. For the univariate meta-regression analysis, publication year (35.7%) and the proportion of female patients (43.6%) seemed to be responsible for the heterogeneity in the effect of VR on preoperative anxiety. Multivariate meta-regression analysis identified the total proportion of female patients as an independent variable, accounting for the considerable level of heterogeneity (65.4%). A symmetrical funnel plot revealed a low risk of publication bias (Figure 3).

### 3.3. Satisfaction and Behavior Disturbance

**Figure 4:**
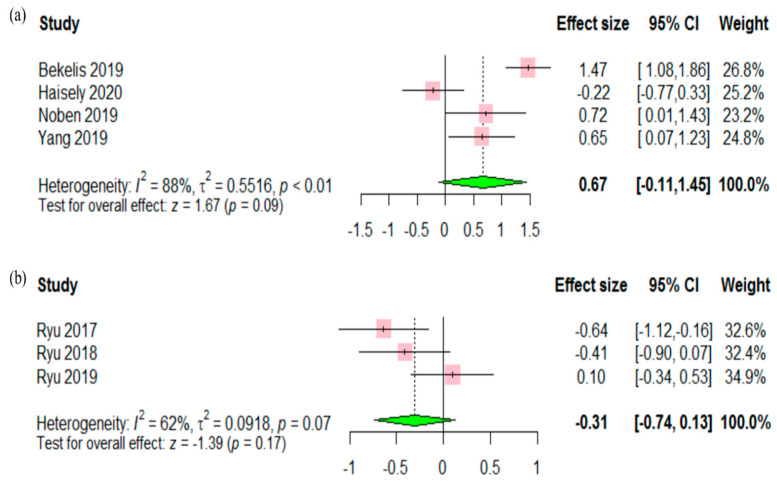
Forest plot comparing (**a**) satisfaction score and (**b**) behavior disturbance between the VR group and control group. Significant differences were not observed between the two groups.

Satisfaction was reported in four RCTs, which included a total of 312 adult patients [21,24,25,30]. Significant difference was not found on postoperative satisfaction between the two groups (SMD 0.67, 95% CI −0.11 to 1.45, *p* = 0.09) (Figure 4a). Behavior disturbance was reported in three RCTs, which included a total of 218 pediatric patients [27,28,29]. A statistically significant difference was not observed on behavior disturbance between the two groups (SMD −0.31, 95% CI −0.74 to 0.13, *p* = 0.17) (Figure 4b).

### 3.4. Risk of Bias

**Figure 5:**
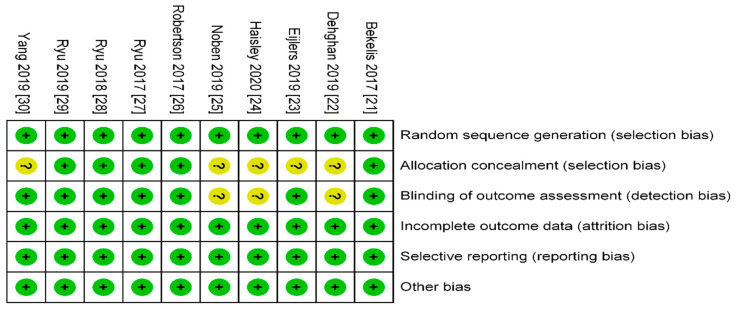
Risk of bias graph. The authors’ judgement about each risk of bias item for each included study. Abbreviations: + = low risk of bias, ? = unclear risk of bias.

The risk of bias is shown in Figure 5 and the reasons for the judgement of each bias are summarized in Table S2. Patients were randomly allocated to each group in all RCTs. Allocation concealment was adequate in five RCTs [21,26,27,28,29] and unclear in the other five RCTs [22,23,24,25,30]. The risk of detection bias was considered as low in seven RCTs [21,23,26,27,28,29,30]. All RCTs were well controlled for attrition, reporting, and other biases.

## 4. Discussion

The present study demonstrates that VR is an effective strategy for relieving preoperative anxiety. It is interesting to note that the efficacy of VR on preoperative anxiety appears to be more prominent in pediatric patients than in adult patients. However, the findings indicate that VR may not improve satisfaction or behavior disturbance after surgery.

Preoperative anxiety scores were significantly lower in the VR group than in the control group. This supports previous findings in the literature. Our previous study investigating pediatric patients who received VR prior to undergoing chest radiography showed that VR decreased the number of distressed patients from 48% to 22.4% [32]. Ganry et al. [33] measured the salivary cortisol concentration, which is a biological marker of anxiety, in 20 patients scheduled for surgery. They found a significant reduction in salivary cortisol concentration after VR application. VR provides patients with visual and auditory senses using several devices (head-mounted displays, smartphones, or headphones) [34]. Those sensory inputs may attenuate preoperative anxiety by two mechanisms. First, in a virtual word, patients can move around the operating room and experience the surgical environment in advance. Patients feel familiar with this surgical environment and predict upcoming events [8,9]. This improves the patient’s ability to predict and deal with the stressful future event [35]. These processes of familiarization and prediction, so-called habituation, may result in effective extinction in fear responses [10,36]. In addition, patients may sense the difference between imaginary fear and reality [10]. Second, VR can divert patient’s attention with attractive audiovisual materials, which dispels patient’s preoperative anxiety [12,37]. Although the benefits of VR over other non-pharmacological interventions with respect to the effect on preoperative anxiety remains unclear, a recent meta-analysis insisted that VR may be more effective than other non-pharmacological tools [13]. Comparing the results of previous meta-analyses, the effect size of VR on anxiety was higher than that of music or games on anxiety (1.32 vs. 0.61 or 0.35) [13,38,39]. Nonetheless, further research is warranted to better establish the advantage of VR over other methods.

It is notable that VR significantly alleviated preoperative anxiety in pediatric patients, while VR was more or less ineffective in adult patients. This is in line with previous results showing that VR interventions for anxiety may be more helpful in younger children [40]. This can be attributed to higher levels of anxiety in pediatric patients [40]. Pediatric patients fear unfamiliar environments, strangers, and odd sounds more so than adult patients [41,42]. In all RCTs included in this meta-analysis [22,23,27,28,29], VR exposed pediatric patients to scheduled situations and enabled them to be familiar with the operating room. Another possible reason for the finding may be that pediatric patients were generally more engaged in and captivated by audiovisual contents [43,44].

The result of this study showed no significant differences on secondary outcomes—satisfaction and behavior disturbance—between the VR and control groups. Additionally, the CI of secondary outcomes was wide and contained the null hypothesis value. This may be interpreted as the present study failing to draw a clear conclusion on the topic of secondary outcomes. Further large-scaled studies are needed to establish the long term effect of VR.

The findings of this meta-analysis should be interpreted with caution. First, a limited number of studies (*n* = 10) were included in the final analysis and the findings of secondary outcomes are based on three or four RCTs. The DerSimonian-Laird approach, a conventional method, was used to estimate the effect size in this study. However, several studies mention the poor performance of the DerSimonian-Laird approach for meta-analyses with a small number of studies [45,46]. Instead, likelihood or empirical Bayesian methods were recommended to guarantee reliable conclusions [45,46]. We performed additional meta-analyses using both likelihood and empirical Bayesian methods to overcome the shortcomings of the DerSimoniam-Laird approach and confirmed the robustness of the current findings. The results of likelihood or empirical Bayesian methods are shown in Supplementary data 1. Second, considerable heterogeneity (80–90% of I^2^) was found among the studies and overall claims with respect to the effect of VR could be weakened. The possible explanation for this heterogeneity may be explained by the different mechanisms of VR. As mentioned above, the mechanisms of VR consist of exposure or distraction. In this meta-analysis, VR was used as exposure therapy in eight RCTs [21,22,23,25,27,28,29,30] and as a distraction technique in two RCTs [24,26]. Third, the total proportions of female patients accounted for heterogeneity according to the results of the meta-regression analysis. Similarly, the effect of VR on anxiety differed by gender in previous studies, including adult patients who were undergoing dental procedures [47]. Fourth, preoperative anxiety was measured using various types of questionnaires. Nowadays, several validated tools are used to evaluate anxiety [48]. A total of six of these tools were used in the 10 RCTs included in this meta-analysis; self-reported tools were used for adult patients, while observational instruments were used for pediatric patients. Finally, various types of surgery were included in this study and seemed to affect the level of preoperative anxiety.

Whether presurgical VR treatment can reduce anesthetic needs, reduce postoperative pain, reduce analgesia requirements, and/or can improve the quality of recovery in patients undergoing surgery are important topics for future research.

## 5. Conclusions

In conclusion, this meta-analysis validated the effectiveness of VR on reducing preoperative anxiety in pediatric patients. However, the effect of VR on postoperative satisfaction or behavior disturbance remains to be clarified.

## Figures and Tables

**Table 1:** Characteristics of included randomized controlled trials.

Author	Sample Size (VR/Control)	Age		Anxiety Measure	Surgery
		VR	Control		
Bekelis 2017 [21]	64/63	57.3	53.4	APAIS	Craniotomy or spine surgery
Dehghan 2019 [22]	20/20	7.35		YPAS	Abdominal surgery
Eijlers 2019 [23]	94/97	8.3	7.5	mYPAS	Maxillofacial, dental, Ear-Nose-Throat surgery
Haisley 2020 [24]	26/26	65.5	61.5	NRS	Minimally invasive foregut
Noben 2019 [25]	49/48	32.6	33.12	VAS	Cesarean delivery
Robertson 2017 [26]	20/20	47		HADS	Arthroscopic knee surgery
Ryu 2017 [27]	34/35	6	6	mYPAS	Elective surgery
Ryu 2018 [28]	34/35	5	6	mYPAS	Elective day surgery
Ryu 2019 [29]	41/39	6	6	mYPAS	Elective surgery
Yang 2019 [30]	24/24	32.5	38.0	APAIS	Arthroscopic knee surgery

Age is expressed as the mean or median values. VR = virtual reality, APAIS = Amsterdam Preoperative Anxiety and Information score, YPAS = Yale Preoperative Anxiety Scale, mYPAS = modified Yale Preoperative Anxiety Scale, NRS = Numeric Rating Scale, VAS = Visual Analogue Scale, HADS = Hospital Anxiety and Depression Scale.

**Table 2:** Meta-regression for the potential sources of heterogeneity.

Variables	Univariate			Multivariate		
	OR	95% CI	*p* Value	OR	95% CI	*p* Value
Age	0.00	−0.02 to 0.02	0.97			
Female	2.07	0.46 to 3.69	0.01	1.63	0.21 to 3.05	0.02
Sample size	0.00	−0.01 to 0.01	0.82			
Publication year	0.39	0.03 to 0.75	0.03	0.28	−0.03 to 0.58	0.07
Pediatric	−0.15	−1.09 to 0.80	0.76			

OR = odds ratio, CI = confidence interval.

## Supplementary Materials

The following are available online at https://www.mdpi.com/2077-0383/9/10/3151/s1, Table S1: Search strategies for each database, Table S2: Details of judgement for each risk of bias for randomized controlled trials, Figure S1: Sensitivity analysis of preoperative anxiety, Figure S2: Cumulative meta-analysis of preoperative anxiety. Supplementary data 1: The results of likelihood or empirical Bayesian method.
