# Supplementary material for: The Effect of Virtual Reality on Preoperative Anxiety: A Meta-Analysis of Randomized Controlled Trials
Source: J Clin Med. 2020 Sep 29;9(10):3151. doi: 10.3390/jcm9103151 (PMC7600416; doi:10.3390/jcm9103151)
Supplement: Supplementary file 1 [file jcm-09-03151-s001.pdf]

Table S1. Search strategies for each database.

Database	Order	Keywords	Results
PubMed	#1	("elective surgical procedures"[MH] OR surgery[MH] OR surgery[TIAB]) OR ("Anesthesia"[MH] OR anesthesia[TIAB] OR anaesthesia[TIAB] OR anesth*[TIAB] OR anaesth*[TIAB])	3927681
	#2	"virtual reality"[MH] OR "virtual reality"[TIAB]	10378
	#3	#1 AND #2	2580
	#4	#3 AND HSSS(S)	732
EMBASE	#1	Surgery/exp OR surgery:ab,ti OR anesthesia:exp OR anesthesia:ab,ti OR anaesthesia:ab,ti OR anesth*:ab,ti OR anaesth*:ab,ti	5726915
	#2	'virtual reality'/exp OR 'virtual reality':ab,ti	20579
	#3	#1 AND #2	5273
	#4	'crossover procedure'/exp OR 'crossover procedure' OR 'double blind procedure'/exp OR 'double blind procedure' OR 'randomized controlled trial'/exp OR 'randomized controlled trial' OR 'single blind procedure'/exp OR 'single blind procedure' OR random* OR factorial* OR crossover* OR 'cross over' OR 'cross-over' OR placebo* OR (doubl* AND blind*) OR (singl* AND blind*) OR assign* OR allocat* OR volunteer*	2587811
	#5	#3 AND #4	865
	#6	#5 AND [embase]/lim	786
CENTRAL	#1	[mh "elective surgical procedures"] OR [mh "general surgery"] OR surgery:ti,ab,kw OR [mh anesthesia] OR anesthesia:ti,ab,kw OR anaesthesia:ti,ab,kw OR anesth*:ti,ab,kw OR anaesth*:ti,ab,kw	237521
	#2	[mh "virtual reality"] OR "virtual reality":ti,ab,kw	2932
	#3	#1 AND #2	509
	#4	#3 AND Trials	506
CINAHL	S1	MH(Surgery, Elective+)	1944
	S2	MH(Surgery, Operative+)	211371
	S3	TI (surgery) OR AB(surgery)	79736
	S4	MH(anesthesia+) OR TI(anesth*) OR AB(anesth*) OR TI(anaesth*) OR AB(anaesth*)	21598
	S5	S1 OR S2 OR S3 OR S4	254624
	S6	MH(Virtual Reality+) OR TI(virtual reality) OR AB(virtual reality)	3041
	S7	S5 AND S6	542
	S8	MH(Clinical Trials) OR PT(Clinical trial) OR TX (clinic* n1 trial*) OR TX ((singl* n1 blind*) OR (singl* n1 mask*)) OR TX ((doubl* n1 blind*) OR (doubl* n1 mask*)) OR TX ((tripl* n1 blind*) OR (tripl* n1 mask*)) OR TX ((trebl* n1 blind*) or (trebl* n1 mask*)) OR TX (randomi* control* trial*) OR MH(Random Assignment) OR TX(random* allocat*) OR TX(placebo*)	586436
	S9	S7 AND S8	106
Scopus	#1	(INDEXTERMS(elective surgical procedures) OR INDEXTERMS(surgery) OR TITLE-ABS(sugery)) OR (INDEXTERMS(anesthesia) OR TITLE-ABS(anesthesia) OR TITLE-ABS(anaesthesia) OR TITLE-ABS(anesth*) OR TITLE-ABS(anaesth*)	1857596
	#2	INDEXTERMS(virtual reality) OR TITLE-ABS(virtual reality)	128389
	#3	#1 AND #2	5215
	#4	(INDEXTERMS(randomized controlled trial) OR INDEXTERMS(controlled clinical trial) OR TITLE-ABS(randomized) OR TITLE-ABS(placebo) OR INDEXTERMS(drug therapy) OR TITLE-ABS(randomly) OR TITLE-ABS(trial) OR TITLE-ABS(groups)) AND NOT (INDEXTERMS(animals) AND NOT INDEXTERMS(humans))	9284998
	#5	#3 AND #4	965
Web of Science	#1	TS=(surgery OR anesthesia OR anaesthesia OR anesth* OR anaesth*)	1378220
	#2	TS=("virtual reality")	21372
	#3	#1 AND #2	2723
	#4	TS=(random* OR blind* OR allocat* OR assign* OR trial* OR placebo* OR crossover OR cross-over* OR intervention)	4147905
	#5	#3 AND #4	869

Table S2. Details for judgement for each risk of bias for randomized controlled studies.

Study	Bias	Author's judgement	Reason for judgement
Bekelis 2017	Random sequence generation (selection bias)	Low	A block randomization design was used with randomly permuted block sizes of 4 on the basis of a computerized random-number generator with sequentially numbered opaque, sealed envelopes for each stratum.
	Allocation concealment (selection bias)	Low	
	Blinding (detection bias)	Low	The physicians conducting the interviews to collect the primary and secondary outcome data, and the data analysts were blinded to the group assignments.
	Incomplete outcome data (attrition bias)	Low	Some patients were excluded from the final analysis in both groups, but reasons for this are both reported and balance across groups.
	Selective reporting (reporting bias)	Low	All pre-specified and expected outcomes are reported.
	Other bias	Low	No other bias was detected.
Dehghan 2019	Random sequence generation (selection bias)	Low	Allocations to groups were performed randomly by assigning patients with even hospital bed numbers in the interventional group and those with odd hospital bed numbers in the control group.
	Allocation concealment (selection bias)	Unclear	Allocations seemed to be unconcealed.
	Blinding (detection bias)	Unclear	Not described
	Incomplete outcome data (attrition bias)	Low	All patients completed the study and there were no losses.
	Selective reporting (reporting bias)	Low	All pre-specified and expected outcomes are reported.
	Other bias	Low	No other bias was detected.
Eijlers 2019	Random sequence generation (selection bias)	Low	Block randomization was performed, stratified by type of surgery.
	Allocation concealment (selection bias)	Unclear	Not described
	Blinding (detection bias)	Low	Assessments after randomization were performed by the blinded researcher, blinded recovery nurse.
	Incomplete outcome data (attrition bias)	Low	Patients in VR group discontinued intervention, but the outcomes were analyzed as intention-to-treat.
	Selective reporting (reporting bias)	Low	All pre-specified and expected outcomes are reported.
	Other bias	Low	No other bias was detected.
Haisley 2020	Random sequence generation (selection bias)	Low	Patients were then randomized via computer software to the VR or the non-VR arm.
	Allocation concealment (selection bias)	Unclear	Not described
	Blinding (detection bias)	Unclear	Not described
	Incomplete outcome data (attrition bias)	Low	All patients completed the study and there were no losses.

	Selective reporting (reporting bias)	Low	All pre-specified and expected outcomes are reported.
	Other bias	Low	No other bias was detected.
Noben 2019	Random sequence generation (selection bias)	Low	Randomization was performed by the researcher using a Web-based computer randomizer generating a randomization list. Randomization blocks of 10 were used.
	Allocation concealment (selection bias)	Unclear	Not described
	Blinding (detection bias)	Unclear	Not described
	Incomplete outcome data (attrition bias)	Low	Outcomes were reported for all patients.
	Selective reporting (reporting bias)	Low	All pre-specified and expected outcomes are reported.
	Other bias	Low	No other bias was detected.
Robertson 2017	Random sequence generation (selection bias)	Low	They were then randomly allocated into one of three groups using a random number generator.
	Allocation concealment (selection bias)	Low	Patient randomization numbers were concealed in opaque envelopes that were opened by the study investigator
	Blinding (detection bias)	Low	Participants were not made aware of their allocated group until after their response to the initial HADS.
	Incomplete outcome data (attrition bias)	Low	Outcomes were reported for all patients.
	Selective reporting (reporting bias)	Low	All pre-specified and expected outcomes are reported.
	Other bias	Low	No other bias was detected.
Ryu 2017	Random sequence generation (selection bias)	Low	Randomization was performed by an independent anaesthetist not otherwise involved in the trial.
	Allocation concealment (selection bias)	Low	A computer-generated randomization code (Random Allocation Software version 1.0) with opaque envelopes containing sequential numbers was used.
	Blinding (detection bias)	Low	A single-blinded assessor collected the observational scores to maintain consistency and uniformy.
	Incomplete outcome data (attrition bias)	Low	One patient discontinued intervention due to acceptable reason.
	Selective reporting (reporting bias)	Low	All pre-specified and expected outcomes are reported.
	Other bias	Low	No other bias was detected.
Ryu 2018	Random sequence generation (selection bias)	Low	Using a computer-generated randomization code, the enrolled patients were randomly allocated to one of two groups.
	Allocation concealment (selection bias)	Low	An opaque envelope containing sequential numbers
	Blinding (detection bias)	Low	All outcomes were assessed by a blind single evaluator
	Incomplete outcome data (attrition bias)	Low	One patient allocated to the VR group was excluded due to acceptable reason.
	Selective reporting	Low	All pre-specified and expected outcomes are reported.

	(reporting bias)		
	Other bias	Low	No other bias was detected.
Ryu 2019	Random sequence generation (selection bias)	Low	A computer-generated randomized code with nontransparent envelopes that contained sequential numbers was used.
	Allocation concealment (selection bias)	Low	
	Blinding (detection bias)	Low	The outcome assessor and an anesthesiologist were blinded to group assignment.
	Incomplete outcome data (attrition bias)	Low	Three patients were losses to follow-up, but acceptable reason was described.
	Selective reporting (reporting bias)	Low	All pre-specified and expected outcomes are reported.
	Other bias	Low	No other bias was detected.
Yang 2019	Random sequence generation (selection bias)	Low	Patients were randomized into 2 groups using a computer-generated random sequence.
	Allocation concealment (selection bias)	Unclear	Not described
	Blinding (detection bias)	Low	The single assessor collecting the outcome data was blinded to the study group assignments.
	Incomplete outcome data (attrition bias)	Low	All patients completed the study and there were no losses.
	Selective reporting (reporting bias)	Low	All pre-specified and expected outcomes are reported.
	Other bias	Low	No other bias was detected.

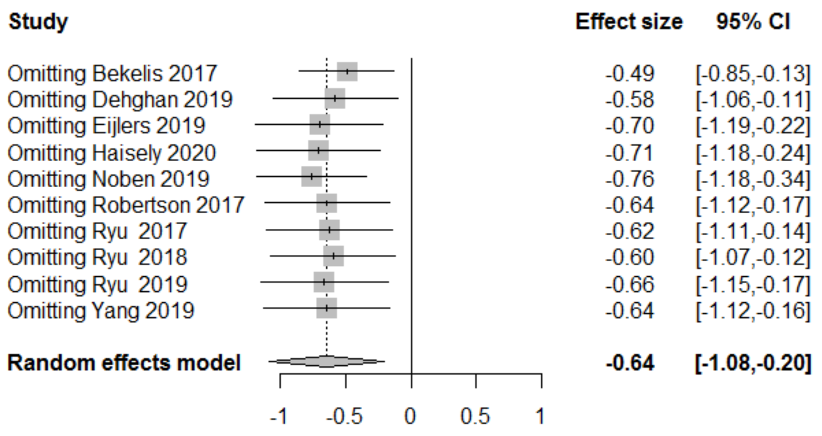

Figure S1. Sensitivity analysis of preoperative anxiety.

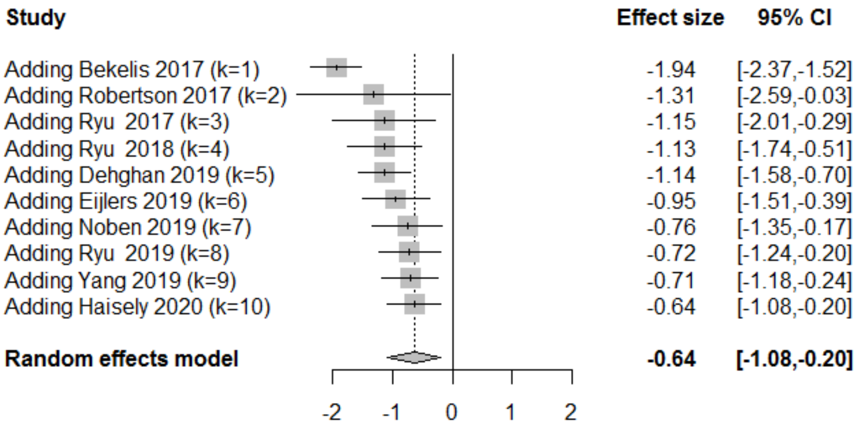

Figure S2. Cumulative meta-analysis of preoperative anxiety.

Supplementary data 1: The results of likelihood or empirical Bayesian method.

1. Likelihood based model

A) Preoperative anxiety

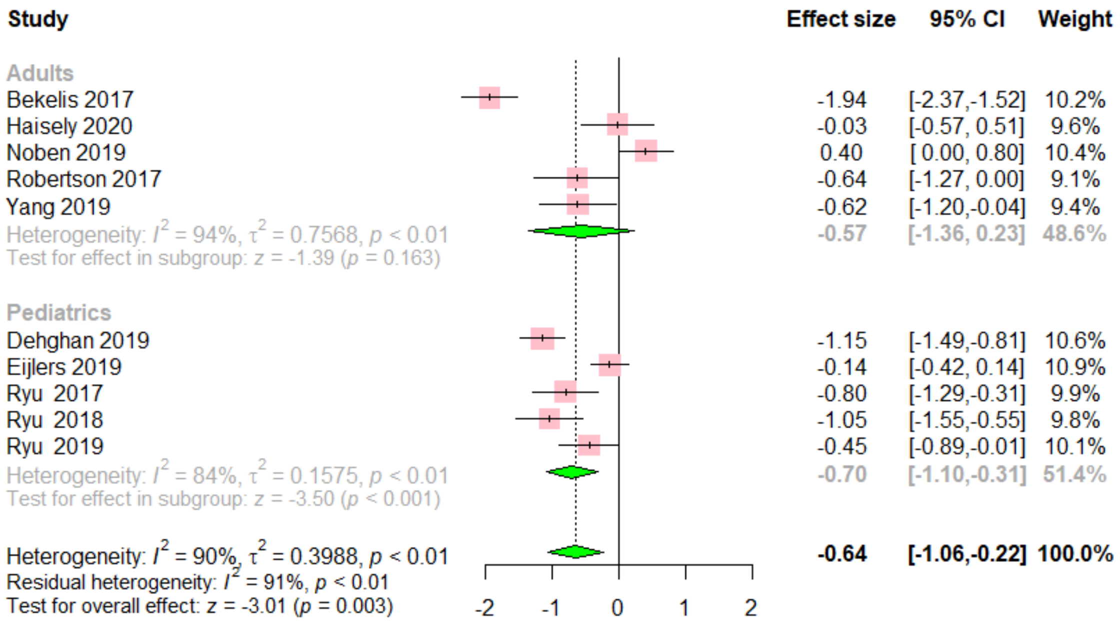

B) Satisfaction

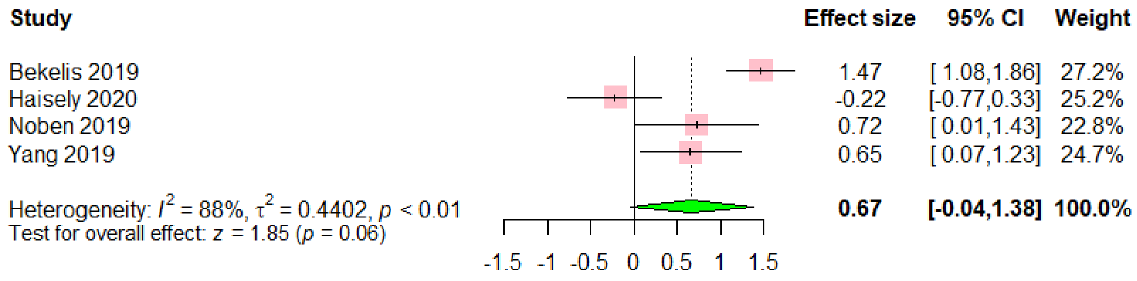

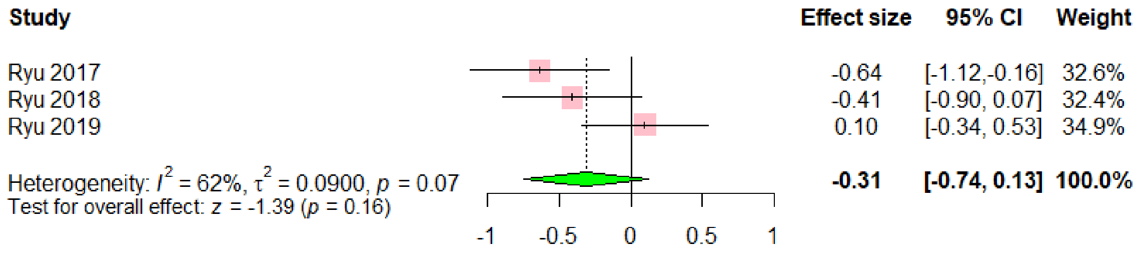

C) Behavior disturbances

2. Empirical Bayesian methods

A) Preoperative anxiety

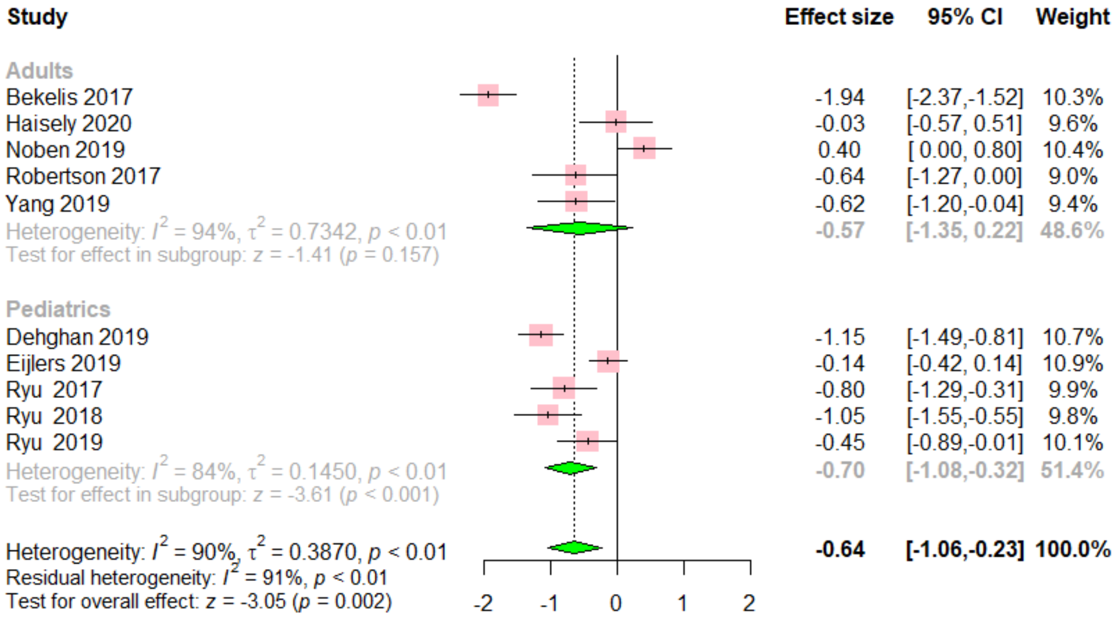

B) Satisfaction

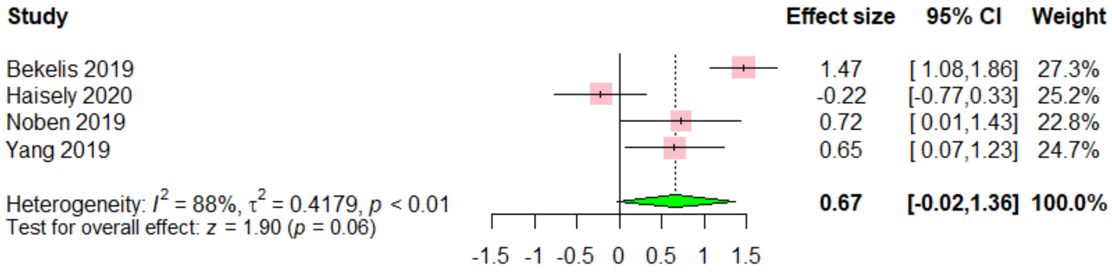

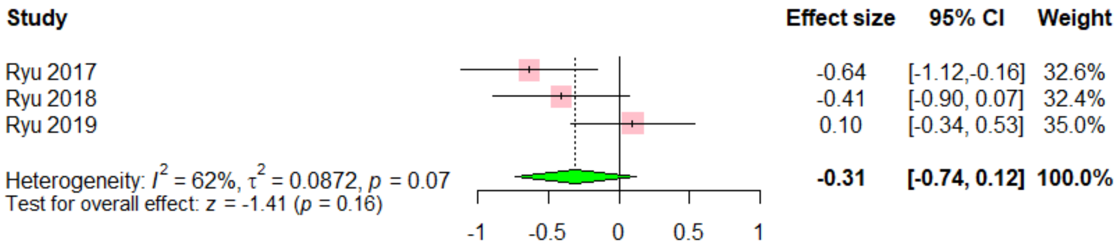

C) Behavior disturbances
